# IGF2BP1-regulated expression of ERRα is involved in metabolic reprogramming of chemotherapy resistant osteosarcoma cells

**DOI:** 10.1186/s12967-022-03549-7

**Published:** 2022-08-02

**Authors:** Qing He, Peng Hao, Gang He, Hantao Mai, Wenzhou Liu, Weiqiong Zhang, Kelin Zhang, Guifang Zhong, Ruilian Guo, Changzhi Yu, Yang Li, Chipiu Wong, Qian Chen, Yantao Chen

**Affiliations:** 1 Department of Surgical Intensive Care Unit, Sun Yat-sen Memorial Hospital, Sun Yat-sen University, Guangzhou, China; 2 Department of Orthopedics, Guangzhou Zengcheng District People’s Hospital, Guangzhou, China; 3 Department of Orthopedics, Sun Yat-sen Memorial Hospital, Sun Yat-sen University, No.107, Yanjiang West Road, Yuexiu, Guangzhou, 510120 China; 4 Department of Chinese Traditional Medicine, Sun Yat-sen Memorial Hospital, Sun Yat-sen University, Guangzhou, China; 5 Pediatric Hematology & Oncology, Sun Yat-sen Memorial Hospital, Sun Yat-Sen University, Guangzhou, China

**Keywords:** Insulin-like growth factor 2 mRNA binding protein 1, Estrogen-related receptor alpha, Osteosarcoma, Doxorubicin, N^6^‑methyladenine

## Abstract

Doxorubicin (Dox) is the standard treatment approach for osteosarcoma (OS), while acquired drug resistance seriously attenuates its treatment efficiency. The present study aimed to investigate the potential roles of metabolic reprogramming and the related regulatory mechanism in Dox-resistant OS cells. The results showed that the ATP levels, lactate generation, glucose consumption and oxygen consumption rate were significantly increased in Dox-resistant OS cells compared with parental cells. Furthermore, the results revealed that the increased expression of estrogen-related receptor alpha (ERRα) was involved in metabolic reprogramming in chemotherapy resistant OS cells, since targeted inhibition of ERRα restored the shifting of metabolic profiles. Mechanistic analysis indicated that the mRNA stability, rather than ERRα transcription was markedly increased in chemoresistant OS cells. Therefore, it was hypothesized that the 3ʹ-untranslated region of ERRα mRNA was methylated by N^6^-methyladenine, which could further recruit insulin-like growth factor 2 mRNA binding protein 1 (IGF2BP1) to suppress mRNA decay and increase mRNA stability. IGF2BP1 knockdown downregulated ERRα and reversed the metabolic alteration of resistant OS cells. Additionally, the oncogenic effect of the IGF2BP1/ERRα axis on Dox-resistant OS cells was verified by in vitro and in vivo experiments. Clinical analysis also revealed that the expression levels of IGF2BP1 and ERRα were associated with the clinical progression of OS. Collectively, the current study suggested that the IGF2BP1/ERRα axis could regulate metabolic reprogramming to contribute to the chemoresistance of OS cells.

## Introduction

Osteosarcoma (OS) is characterized by increased metastatic and aggressive potential in children and young individuals [1]. Doxorubicin (Dox) chemotherapy combined with cisplatin (CDDP) is a standard therapeutic approach for treating patients with OS [2]. However, the acquired drug-resistance can seriously attenuate the effectiveness of chemotherapy [3]. It has been reported that several mechanisms such as drug inactivation by glutathione S-transferase P1, increased apoptosis and autophagy, and the dysregulation of epigenetic regulators can be involved in the resistance OS cells to chemotherapy [4]. Since enhanced chemosensitivity can significantly improve the survival rate of patients with OS, studies investigating the particular mechanisms involved in the resistance of OS cells to chemotherapy are desperately required.

The association between metabolic reprogramming and cancer, including chemoresistance, has attracted increasing attention [5]. It has been previously reported that glycolysis not only provides energy for cell proliferation and growth, but also triggers chemoresistance in several types of cancer, including OS [6]. The alteration of mitochondrial metabolism can markedly regulate sensitivity to chemotherapeutic drugs [7]. A previous study demonstrated that estrogen-related receptor alpha (ERRα), a key regulator of mitochondrial biogenesis and energy metabolism, was upregulated in OS cells and tissues [8]. Additionally, a previous study by our laboratory revealed that ERRα was increased in chemoresistant OS cells, while ERRα knockdown, using the corresponding inhibitor or the small interfering (si)RNA technology, could restore the chemosensitivity of OS cells [9]. However, whether metabolic reprogramming, such as glycolysis, contributes to ERRα-regulated OS cell chemoresistance remains unknown. Furthermore, the mechanisms underlying the increased levels of ERRα in chemoresistant OS cells have not been previously illustrated.

N^6^-methyladenosine (m^6^A), the most abundant modification in human mRNA, can regulate mRNA expression via modulating mRNA splicing, decay or translation [10]. For example, a previous study suggested that m^6^A methylated RNA could recruit RNA binding proteins such as insulin-like growth factor 2 mRNA binding proteins (IGF2BPs) and human antigen R to prevent mRNA decay and increase protein production [11]. Additionally, previous studies indicated that m^6^A could accelerate the Warburg effect to trigger the proliferation, growth and metastasis of cancer cells [12, 13]. Furthermore, m^6^A demethylase AlkB homolog 5 modulated casein kinase 2-mediated glycolysis could regulate the sensitivity of bladder cancer cells to cisplatin [14]. Another study demonstrated that IGF2BP1 could specifically bind to the 3′-untranslated region (3′-UTR) of lactate dehydrogenase A (LDHA) mRNA, thus leading to enhanced LDHA mRNA stability and facilitation of glycolysis in colorectal cancer cells [15]. In addition, IGF2BP1 upregulated c-Myc mRNA expression via binding to its 3′-UTR, eventually stabilizing mRNA and promoting glutamine metabolism [16]. Nevertheless, the potential effects of m^6^A on chemoresistance and glycolysis in OS cells remain unknown.

In the present study, the metabolic profiling, including ATP generation, glucose consumption, lactate production and oxygen consumption rate (OCR) was explored in Dox-resistant OS cells. Furthermore, the effect of m^6^A methylation on promoting ERRα expression in chemoresistant OS cells was also investigated.

## Results

### Metabolic reprogramming in Dox-resistant OS cells

**Fig. 1:**
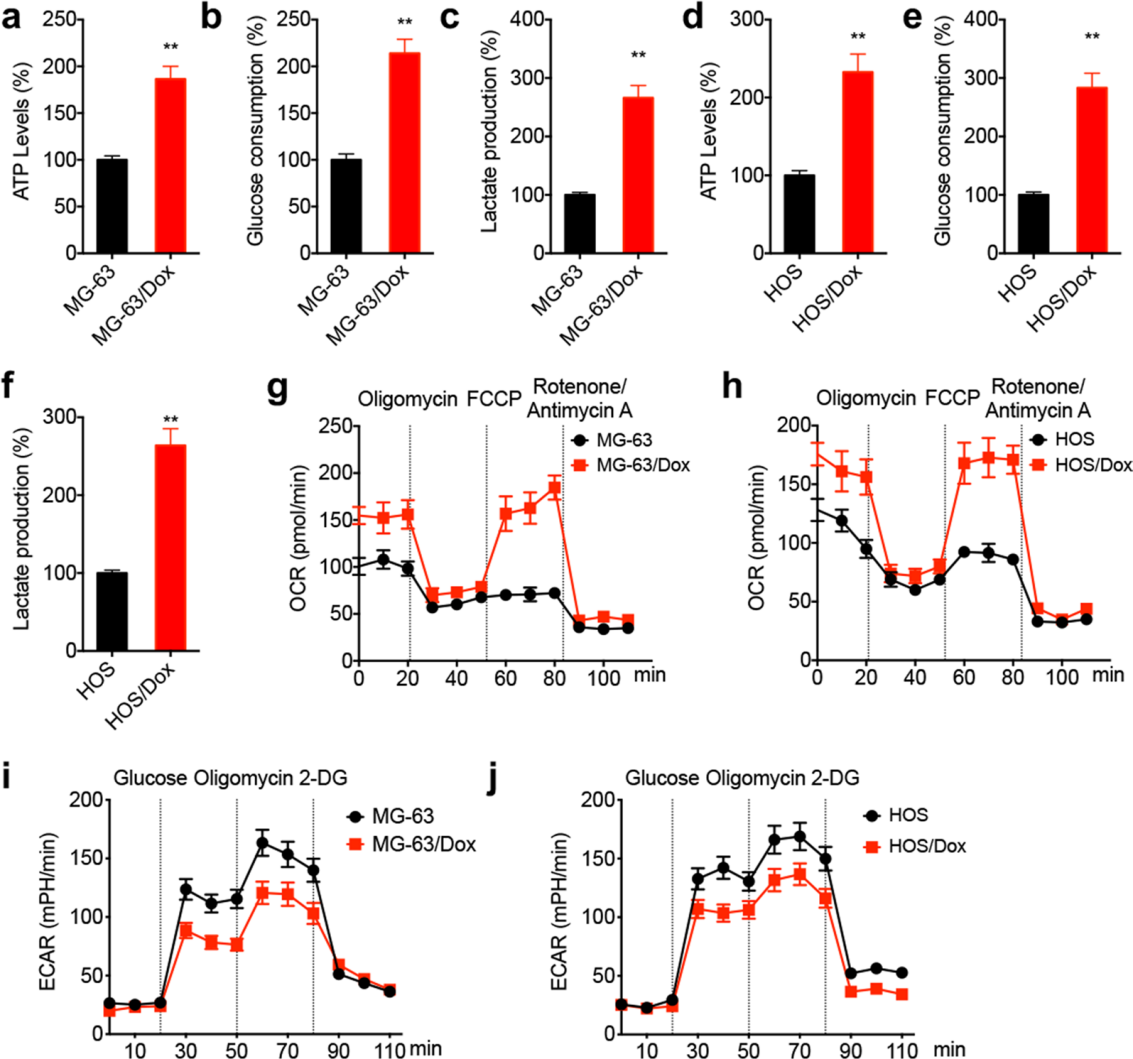
Dox-resistant OS cells showed metabolic reprogramming. The relative levels of ATP generation (**a**), glucose consumption (**b**), and generation of lactate (**c**) in MG-63 and MG-63/Dox cells; The relative levels of ATP generation (**d**), glucose consumption (**e**), and generation of lactate (**f**) in HOS and HOS/Dox cells; Variation of OCR was determined in MG-63/Dox (**g**) and HOS/Dox (**h**) cells and parental cells, respectively, by extracellular flux analysis. Variation of ECAR was determined in MG-63/Dox (**i**) and HOS/Dox (**j**) cells and parental cells, respectively, by extracellular flux analysis. Data are presented as means ± SD from three independent experiments. Each experiment was performed in six replicates for OCR or ECAR analysis. ^**^p < 0.01 by Student’s *t* test

It has been reported that several metabolic processes such as mitochondrial respiration, oxidative phosphorylation and aerobic glycolysis vary in chemoresistant cancer cells [17, 18]. Therefore, herein, the metabolic profile between chemoresistant and parental OS cells was compared. The results showed that compared with MG-63 cells, MG-63/Dox cells displayed increased ATP generation (Fig. 1a), glucose consumption (Fig. 1b) and lactate generation (Fig. 1c). Consistently, HOS/Dox cells also exhibited enhanced ATP generation (Fig. 1d), glucose consumption (Fig. 1e) and lactate generation (Fig. 1f) compared with parental HOS cells. Additionally, Seahorse analysis revealed that the basal and maximal OCR were elevated in Dox-resistant OS cells (Fig. 1g and h) compared with those in parental MG-63 and HOS cells. Furthermore, Dox-resistant OS cells also displayed reduced extracellular acidification rate (ECAR; Fig. 1i and j), thus reflecting the overall glycolytic flux. The above findings demonstrated that Dox-resistant OS cells displayed metabolic reprogramming and enhanced ATP generation.

### ERRα is essential for metabolic reprogramming in Dox-resistant OS cells

**Fig. 2:**
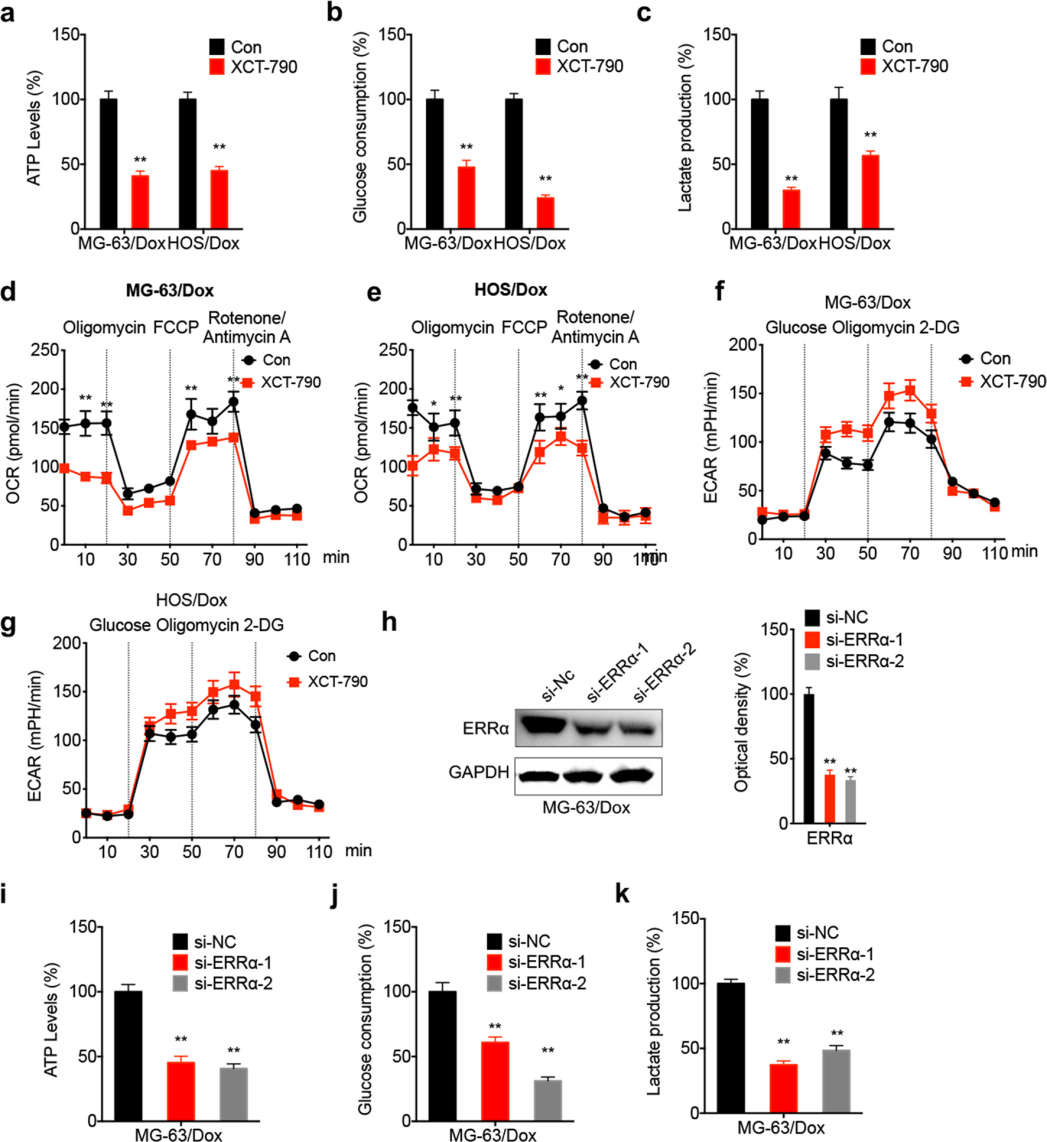
ERRα was essential for metabolic reprogramming in Dox-resistant OS cells. The relative levels of ATP generation (**a**), consumption of glucose (**b**), and production of lactate (**c**) in MG-63/Dox and HOS/Dox cells treated with XCT-790 (1 μM) or vehicle control for 24 h; the variation of OCR in MG-63/Dox (**d**) and HOS/Dox (**e**) cells treated with XCT-790 (1 μM) or vehicle control for 24 h was determined by extracellular flux analysis. The variation of ECAR in MG-63/Dox (**f**) and HOS/Dox (**g**) cells treated with XCT-790 (1 μM) or vehicle control for 24 h was determined by extracellular flux analysis. **h** The protein expression of ERRα in MG-63/Dox cells transfected with si-NC or si- ERRα-1/-2 for 24 h were measured by western blot analysis (left) and quantitively analyzed (right); MG-63/Dox cells were transfected with si-NC or si-ERRα for 24 h, and then the relative levels of ATP generation (**i**), consumption of glucose (**j**), and production of lactate (**k**) were measured. Data are presented as means ± SD from three independent experiments. Each experiment was performed in six replicates for OCR or ECAR analysis. ^**^p < 0.01 by Student’s *t* test

ERRα/β/γ are crucial mediators in cancer cell metabolism [19]. A previous study from our laboratory demonstrated that Dox-resistant OS cells showed increased ERRα levels, thus suggesting that ERRα could regulate the resistance of OS cells in Dox via regulating adenosine triphosphate–binding cassette subfamily B member 1 (ABCB1) expression [9]. Therefore, the current study aimed to investigate whether ERRα could regulate metabolic reprogramming in Dox-resistant OS cells. The results demonstrated that OS cell treatment with XCT-790, an ERRα inverse agonist, significantly attenuated ATP generation (Fig. 2a), glucose consumption (Fig. 2b) and lactate production (Fig. 2c) in Dox-resistant OS cells. Furthermore, exposure of Dox-resistant OS cells to XCT-790 decreased the basal and maximal OCR (Fig. 2d and e), while it enhanced ECAR in both MG-63/Dox (Fig. 2f) and HOS/Dox (Fig. 2g) cells. To verify the essential role of ERRα in metabolic reprogramming in Dox-resistant OS cells, its expression was knocked down following cell transfection with two siRNA clones targeting ERRα (Fig. 2h). The results showed that ATP generation (Fig. 2i), glucose consumption (Fig. 2 j) and lactate production (Fig. 2k) were attenuated in ERRα-depleted MG-63/Dox cells. These results suggested that ERRα could be involved in metabolic reprogramming in Dox-resistant OS cells.

### ERRα mRNA stability is elevated in Dox-resistant OS cells

**Fig. 3:**
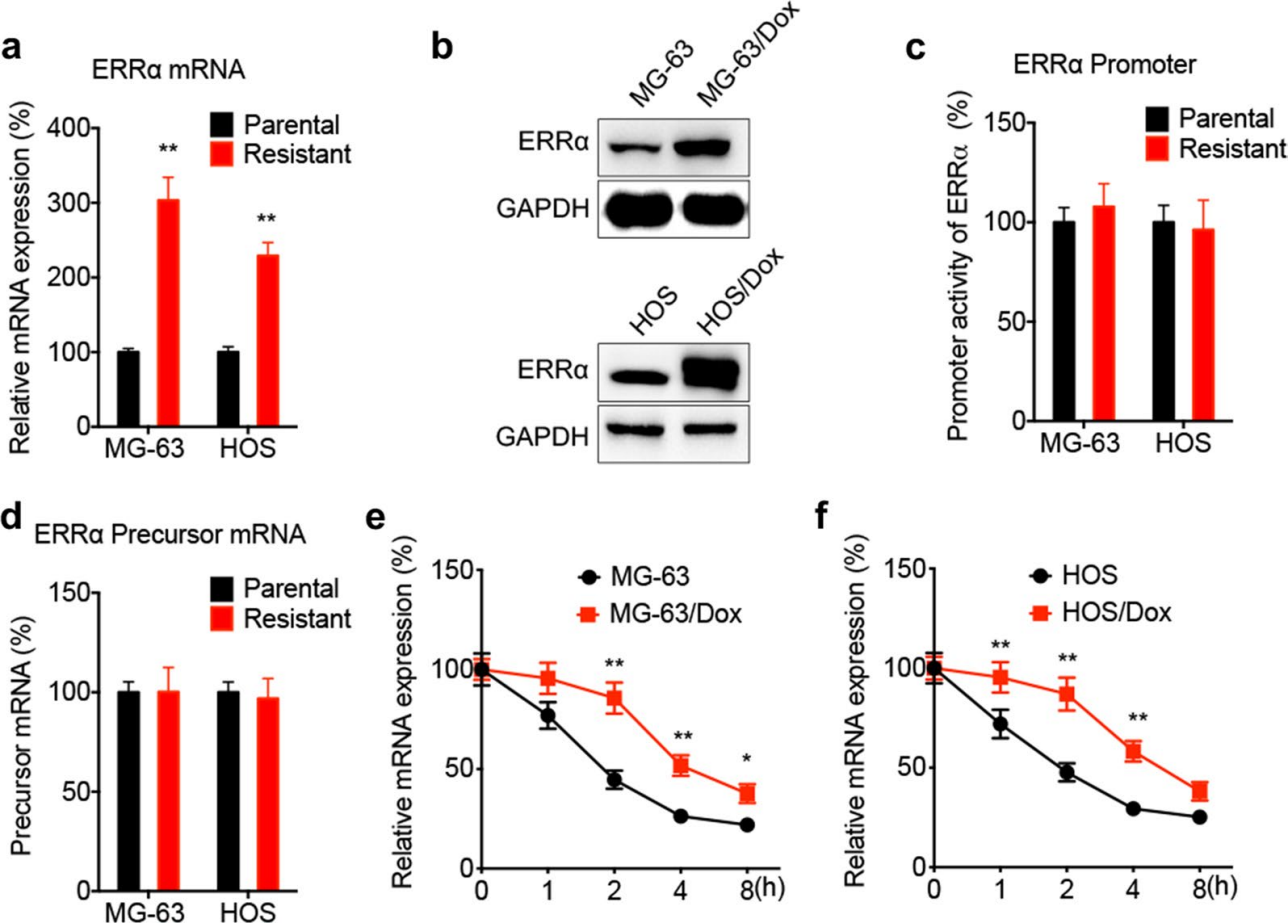
The mRNA stability of ERRα increased in Dox-resistant OS cells. The levels of mRNA (**a**) and protein (**b**) of ERRα in OS parental and Dox-resistant cells; **c** promoter activities in OS parental and Dox-resistant cells pre-transfected with vector control or pGL3-ERRα reporter; **d** the precursor ERRα mRNA in OS parental and Dox-resistant cells were checked by qRT-PCR; **e**, **f** OS parental and Dox-resistant cells were treated with Act-D for different time periods, the mRNA of ERRα was checked by qRT-PCR. Data are presented as means ± SD from three independent experiments. ^**^p < 0.01 by Student’s *t* test

Compared with parental OS cells, the mRNA (Fig. 3a) and protein (Fig. 3b) expression levels of ERRα were increased in Dox-resistant OS cells. However, dual luciferase reporter assays revealed that there was no significant difference in the activity of ERRα promoter between Dox-resistant and parental OS cells (Fig. 3c). Additionally, the levels of precursor ERRα mRNA in Dox-resistant and parental cells were comparable (Fig. 3d). The aforementioned findings suggested that the increased levels of ERRα in Dox-resistant cells could be due to mRNA stabilization. The mRNA stability assay using actinomycin D (Act-D) further verified that the stability of ERRα mRNA was significantly greater in Dox-resistant OS cells compared with parental cells (Fig. 3e and f).

### m^6^A methylated ERRα 3′-UTR serves a critical role in ERRα mRNA stability

**Fig. 4:**
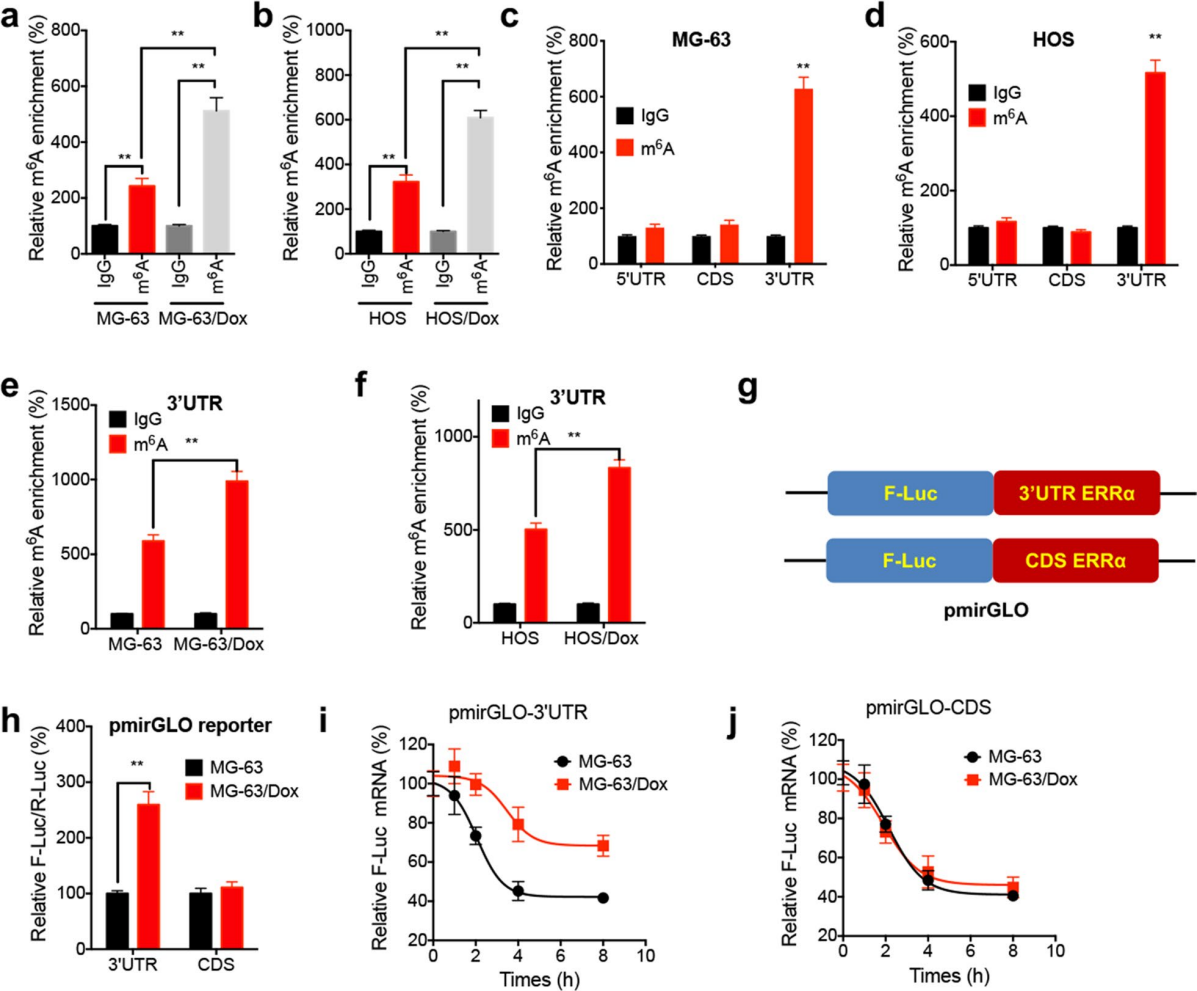
The m^6^A methylated 3′UTR of ERRα was critical for its mRNA stability. The m^6^A methylation of ERRα mRNA in parental or Dox-resistant MG-63 (**a**) and HOS (**b**) cells were checked by m6A-RIP-PCR; the m^6^A enrichment of ERRα mRNA in MG-63 (**c**) or HOS (**d**) cells were checked by m^6^A-RIP-qPCR and fragmented RNA; **e**, **f** The m^6^A methylation of 3′UTR of ERRα mRNA in parental or Dox-resistant MG-63 (**e**) and HOS (**f**) cells; **g** the schematic diagram for luciferase reporter by use of pmirGLO and CDS or 3′UTR of ERRα mRNA; **h** MG-63 or MG-63/Dox cells were transfected with pmirGLO-CDS or pmirGLO-3′UTR for 24 h, and then the relative levels of F-Luc/R-Luc were checked; MG-63 or MG-63/Dox cells were transfected with pmirGLO-CDS (**i**) or pmirGLO-3′UTR (**j**) for 24 h and further incubated with Act-D for different time periods, then the mRNA of F-Luc in cells was checked by qRT-PCR. Data are presented as means ± SD from three independent experiments. ^**^p < 0.01 by Student’s *t* test

A previous study indicated that m^6^A methylation plays a critical role in mRNA stability via recruiting RNA binding proteins [20]. m^6^A-RIP PCR showed that ERRα mRNA was significantly methylated by m^6^A in MG-63 cells, while the enrichment of m^6^A was further increased in MG-63/Dox cells (Fig. 4a). Consistent results were obtained using parental and Dox-resistant HOS cells (Fig. 4b). Additionally, m^6^A-RIP PCR using fragmented RNA revealed that m^6^A was mainly enriched in the 3′-UTR rather than in the coding sequence (CDS) or 5′-UTR of ERRα mRNA in OS cells (Fig. 4c and d). Furthermore, m^6^A enrichment in the 3′-UTR of ERRα mRNA was markedly increased in Dox-resistant OS cells (Fig. 4e and f). To further investigate whether the m^6^A methylated 3’UTR was involved in mRNA stability, a luciferase reporter plasmid was constructed using a pmirGLO vector encompassing the CDS or 3′-UTR sequence of ERRα mRNA (Fig. 4g). The results demonstrated that the activity of F-Luc was notably enhanced in the plasmid encompassing the 3′-UTR rather than the CDS of ERRα mRNA (Fig. 4h). Consistently, the mRNA stability of F-Luc was significantly elevated in 3′-UTR (Fig. 4i), rather than in the CDS (Fig. 4j) of ERRα mRNA.

### IGF2BP1 binds with ERRα 3′-UTR to enhance its mRNA stability

**Fig. 5:**
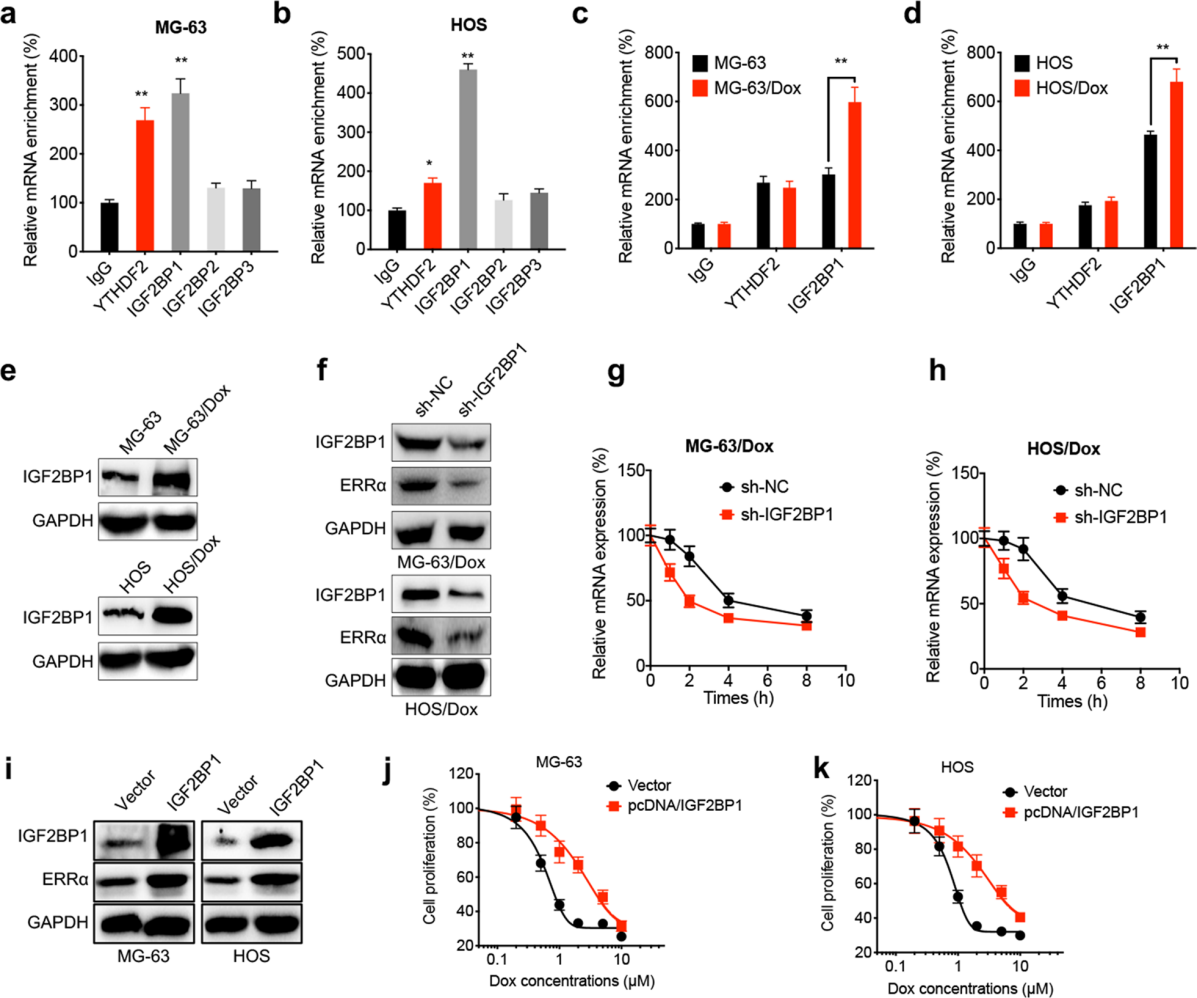
IGF2BP1 binds with 3′UTR of ERRα to increase its mRNA stability. The binding between ERRα mRNA and potential reader proteins in MG-63 (**a**) or HOS (**b**) cells was checked by RIP-PCR; the binding between ERRα mRNA with YTHDF2 or IGF2BP1 in parental or Dox-resistant MG-63 (**c**) and HOS (**d**) cells were checked by RIP-PCR; **e** the expression of IGF2BP1 in Dox-resistant and parental OS cells were checked by western blot analysis; **f** The expression of IGF2BP1 and ERRα in sh-Control and sh-IGF2BP1 Dox-resistant OS cells were checked by western blot analysis; the mRNA of ERRα in sh-Control and sh-IGF2BP1 MG-63/Dox (**g**) or HOS/Dox (**h**) cells treated with Act-D for different time periods; **i** cells were transfected with vector control (pcDNA) or pcDNA/IGF2BP1 for 24 h, and then the expression of IGF2BP1 and ERRα were checked by western blot analysis; MG-63 (**j**) or HOS (**k**) cells were transfected with vector control (pcDNA) or pcDNA/IGF2BP1 for 24 h, and then further treated with increasing concentrations of Dox for 24 h. Data are presented as means ± SD from three independent experiments. ^**^p < 0.01 by Student’s *t* test

It has been reported that the m^6^A-triggered mRNA stability is associated with the recruitment of “reader” proteins, including YTH domain-containing family proteins (YTHDFs), IGF2BPs and YTH domain-containing proteins (YTHDCs) [21]. Since YTHDF2 and IGF2BP1/2/3 serve a critical role in mRNA stability, their roles in m^6^A-triggered ERRα mRNA stability were further explored. RIP-PCR analysis showed that both YTHDF2 and IGF2BP1 could bind with ERRα mRNA in MG-63 and HOS cells (Fig. 5a and b). The binding capacity of IGF2BP1 on ERRα mRNA was enhanced in Dox-resistant MG-63 (Fig. 5c) and HOS cells (Fig. 5d), while that between YTHDF2 and ERRα mRNA remained unchanged. Additionally, IGF2BP1 was upregulated in Dox-resistant cells compared with parental OS cells (Fig. 5e). To further verify the essential role of IGF2BP1, its expression was knocked down in Dox-resistant cells using the short hairpin (sh)RNA technology (Fig. 5f). Therefore, cell transfection with sh-IGF2BP1 notably decreased the protein expression levels of ERRα (Fig. 5f), since IGF2BP1 silencing attenuated ERRα mRNA stability in OS/Dox cells (Fig. 5g and h). By contrast, IGF2BP1 overexpression upregulated ERRα in OS cells (Fig. 5i). Consistently, IGF2BP1 overexpression reduced the sensitivity of both MG-63 (Fig. 5j) and HOS (Fig. 5k) cells to Dox.

### IGF2BP1/ERRα axis regulates metabolic reprogramming in Dox-resistant OS cells

**Fig. 6:**
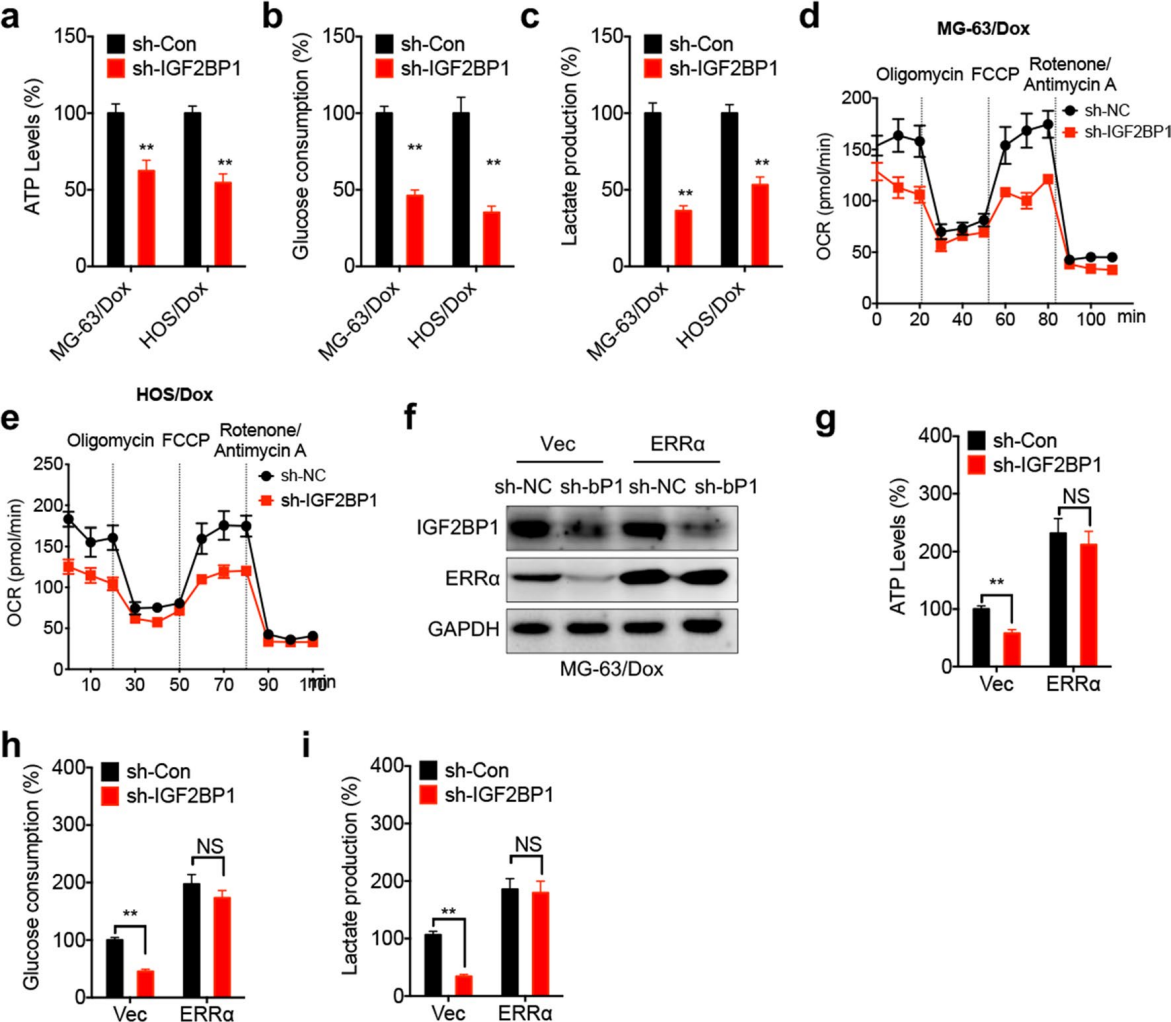
IGF2BP1/ERRα axis regulated metabolic reprogramming of Dox-resistant OS cells. The relative levels of ATP generation (**a**), consumption of glucose (**b**), and production of lactate (**c**) in sh-Control and sh-IGF2BP1 cells; the levels of OCR in sh-Control and sh-IGF2BP1 MG-63/Dox (**d**) or HOS/Dox (**e**) cells were determined by extracellular flux analysis. **f** The protein expression of ERRα in sh-Control or sh-IGF2BP1 cells transfected with vector control or pcDNA/ERRα for 24 h; the ATP generation (**g**), consumption of glucose (**h**), and production of lactate (**i**) in sh-Control and sh-IGF2BP1 MG-63/Dox cell transfected with empty vector or pcDNA/ERRα for 24 h. Data are presented as means ± SD from three independent experiments. Each experiment was performed in six replicates for OCR or ECAR analysis. ^**^p < 0.01, NS, no significant, by Student’s *t* test

The effect of the IGF2BP1/ERRα axis on metabolic reprogramming in Dox-resistant OS cells was further investigated. The results showed that cell transfection with sh-IGF2BP1 reduced ATP generation (Fig. 6a), glucose consumption (Fig. 6b) and lactate production (Fig. 6c) in Dox-resistant OS cells. Additionally, IGF2BP1 silencing decreased the basal and maximal OCR in Dox-resistant OS cells (Fig. 6d and e). However, ERRα overexpression (Fig. 6f) abrogated the IGF2BP1 silencing-mediated suppressed ATP generation (Fig. 6g), glucose consumption (Fig. 6h) and lactate production (Fig. 6i) in MG-63/Dox cells. The aforementioned findings suggested that the IGF2BP1/ERRα axis could regulate metabolic reprogramming in Dox-resistant OS cells.

### IGF2BP1/ERRα axis regulates the sensitivity of OS cells to Dox both in vitro and in vivo

**Fig. 7:**
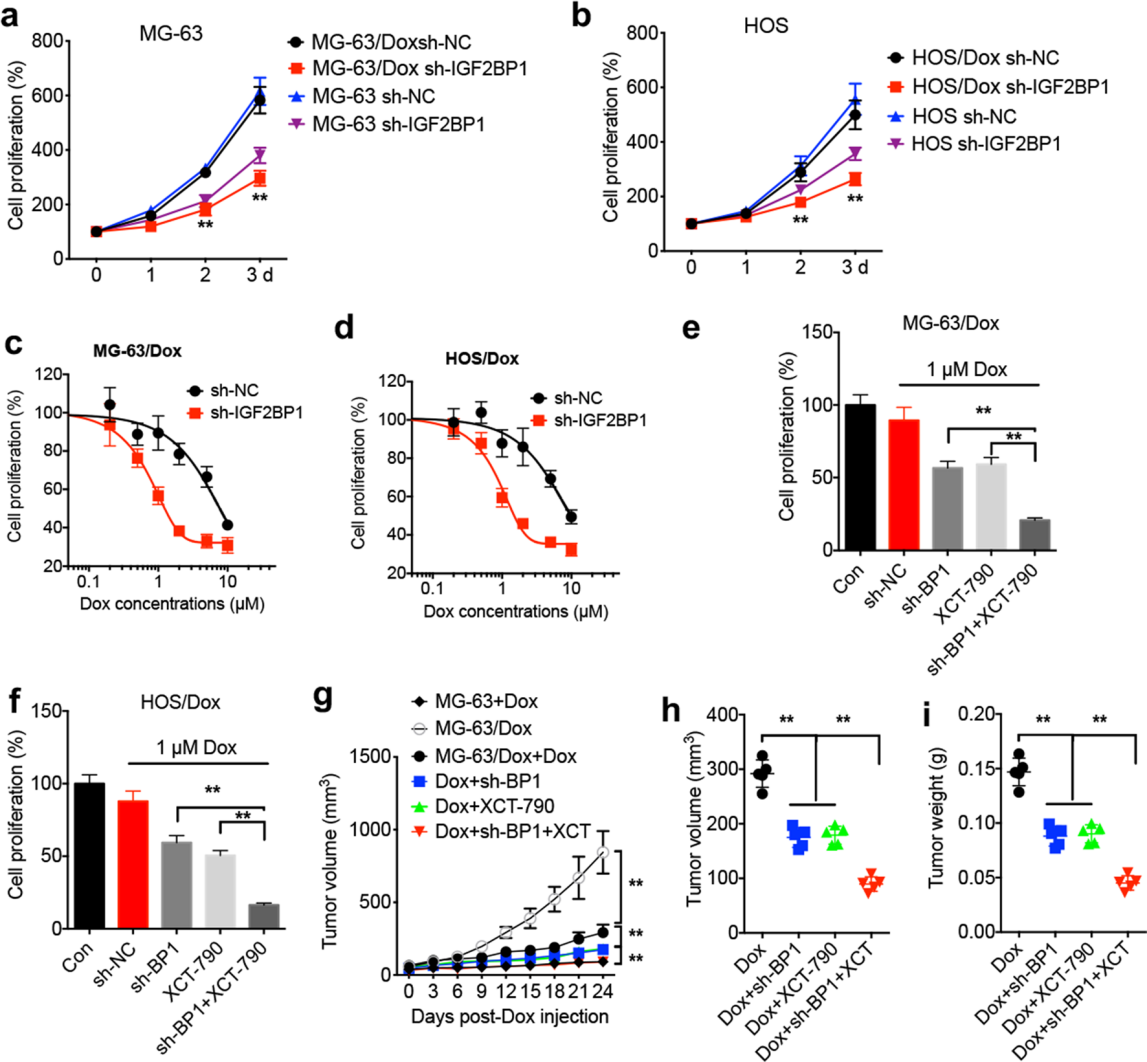
IGF2BP1/ERRα axis regulated in vitro and in vivo Dox sensitivity of OS cells. Cell proliferation of sh-Control and sh-IGF2BP1 MG-63/Dox (**a**) or HSO/Dox (**b**) cells; Dox sensitivity of sh-Control and sh-IGF2BP1 MG-63/Dox (**c**) or HSO/Dox (**d**) cells treated with Dox for 24 h; sh-Control and sh-IGF2BP1 MG-63/Dox (**e**) or HSO/Dox (**f**) cells were treated with 1 μM XCT-790 and 1 μM Dox for 24 h; **g** xenografts of sh-Control and sh-IGF2BP1 MG-63/Dox cells were treated with Dox combined with or without XCT-790. The tumor growth curves were recorded every three days; tumor volume (**h**) and tumor weight (**i**) of xenografts for each group at the end of the experiment. Data are presented as means ± SD from three independent experiments. ^**^p < 0.01 by Student’s *t* test

A previous study from our laboratory suggested that ERRα could be involved in the sensitivity of OS cells to Dox in vitro [9]. Therefore, the current study aimed to evaluate the effects of the IGF2BP1/ERRα axis on Dox sensitivity. The results showed that IGF2BP1 knockdown significantly inhibited the proliferation of MG-63/Dox (Fig. 7a) and HOS/Dox (Fig. 7b) cells, as well as that of parental OS cells. Additionally, IGF2BP1 silencing enhanced the sensitivity of MG-63/Dox and HOS/Dox cells to Dox chemotherapy (Fig. 7c and d). In addition, cell treatment with XCT-790 and IGF2BP1 silencing could synergistically increase the sensitivity of MG-63/Dox and HOS/Dox cells to Dox (Fig. 7e and f). To verify the effects of IGF2BP1 knockdown on Dox sensitivity in vivo, mice were first injected with MG-63/Dox cells transfected with sh-control or sh-IGF2BP1 and were then treated with Dox with or without XCT-790. Both XCT-790 and IGF2BP1 increased Dox sensitivity, while co-treatment of mice with XCT-790 and sh-IGF2BP1 synergistically enhanced the sensitivity of xenografts to Dox in vivo (Fig. 7g). Furthermore, the tumor volume and weight were significantly reduced in the XCT-790 and sh-IGF2BP1 group compared with the XCT-790 or sh-IGF2BP1 groups alone (Fig. 7h and i). These findings indicated that the IGF2BP1/ERRα axis could regulate Dox-resistance in OS both in vitro and in vivo.

### Clinical characteristics of the IGF2BP1/ERRα axis in clinical OS progression

**Fig. 8:**
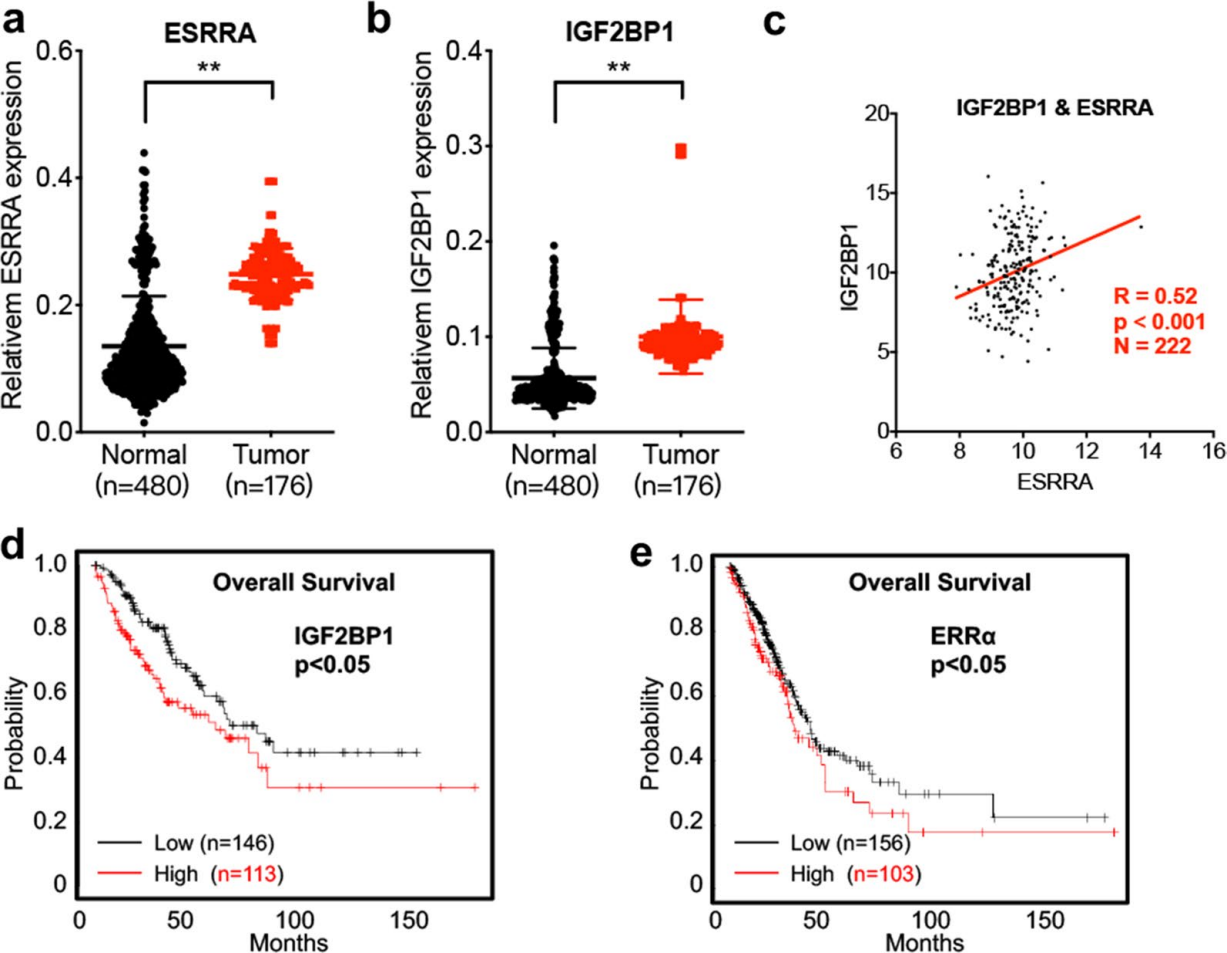
Clinical characteristics of IGF2BP1/ERRα axis on clinical OS progression. Levels of IGF2BP1 (**a**) and ERRα (**b**) in OS tumor and normal tissues with the data from the Oncopression database (https://www.oncopression.com); **c** the Pearson correlation between IGF2BP1 and ERRα in OS tumor tissues on the basis of TCGA data; **d** overall survival in OS patient with high (n = 113) or low (n = 146) protein levels of IGF2BP1. **e** Overall survival in OS patient with high (n = 103) or low (n = 156) protein levels of ERRα

The current study further explored the clinical characteristics of the IGF2BP1/ERRα axis in the clinical progression of OS. Bioinformatics analysis using the Oncopression database (https://www.oncopression.com) predicted that both IGF2BP1 (Fig. 8a) and ERRα (Fig. 8b) were notably upregulated in OS tissues compared with normal ones. Data from The Cancer Genome Atlas (TCGA) predicted that the expression levels of ERRα were markedly associated with those of IGF2BP1 in patients with OS (Fig. 8c). Furthermore, Kaplan–Meier plotter analysis [33] revealed that the increased IGF2BP1 (Fig. 8d) and ERRα (Fig. 8e) expression levels in OS patients were associated with poor overall survival. The above results indicated that the IGF2BP1/ERRα axis exerted an oncogenic role in clinical OS progression.

## Discussion

The acquired resistance to chemotherapeutic drugs such as Dox is considered as the major reason for the failure of OS treatment. It has been reported that metabolic reprogramming such as alterations in glycolysis and the metabolism of glutamine is involved in the resistance of cancer cells to chemotherapy [22]. Herein, compared with OS cells, Dox-resistant OS cells showed increased ATP generation, glucose consumption, lactate production and OCR. A previous study demonstrated that increased glycolysis and tricarboxylic acid cycle could trigger the malignant and aggressive features of OS cells such as cell migration and invasion [23]. Selectively, shifting glycolysis to mitochondrial oxidation can suppress OS cell growth and metastasis [24]. Another study revealed that cisplatin-resistant OS cells showed enhanced glucose metabolism [25]. Additionally, 2-deoxy-d-glucose, a glycolytic inhibitor, could increase the sensitivity of OS cells to paclitaxel [26]. The current and previous studies verified the oncogenic effect of glycolysis on chemoresistant OS cells, thus suggesting that reversing or inhibiting glycolysis could increase the efficiency of clinical chemotherapy.

The results of the present study suggested that ERRα could be a key regulator of Dox-resistance and metabolic reprogramming in OS cells. A previous study from our laboratory revealed that ERRα positively regulated ABCB1 expression to promote cell resistance to Dox [9]. Herein, targeted ERRα silencing attenuated ATP generation, glucose consumption, lactate production and OCR in Dox-resistant OS cells. Emerging evidence has indicated that ERRα regulates the expression of genes involved in glycolysis [27]. For example, ERRα could bind with peroxisome proliferator-activated receptor γ coactivators 1α to increase glucose metabolism in muscles via transcriptionally activating pyruvate dehydrogenase kinase 4 [28]. Furthermore, ERRα could trigger lactate utilization in breast cancer cells to induce cell resistance to targeted therapy [29]. Therefore, inhibition of ERRα could suppress glycolysis in chemoresistant OS cells and overcome resistance to Dox.

Herein, the results showed that the m^6^A methylated 3′-UTR of ERRα and IGF2BP1 recruitment were associated with elevated ERRα mRNA stability in Dox-resistant OS cells. Furthermore, the effect of the IGF2BP1/ERRα axis on maintaining Dox resistance and glucose metabolism were further confirmed. Previous studies demonstrated that IGF2BP1 could bind with m^6^A methylated mRNA to increase mRNA stability and could therefore act as an oncogene [30, 31]. For example, IGF2BP1 upregulated c-Myc mRNA via binding with its 3′-UTR to increase mRNA stability [32]. In OS cells, IGF2BP1 was upregulated in tumor tissues and promoted the growth and metastasis of cancer cells [33, 34]. Consistent with the results of the current study, IGF2BP1 could bind with LDHA 3′-UTR, thus resulting in enhanced LDHA mRNA stability and increased glycolysis in colon cancer cells [15]. Additionally, IGF2BP1 silencing could increase the effectiveness of hyperthermia therapy in colon cancer cells [15]. Herein, ERRα overexpression restored the IGF2BP1 silencing-mediated reduced ATP generation, glucose consumption and lactate production in MG-63/Dox cells, thus suggesting that ERRα could be a downstream effector of the IGF2BP1-regulated metabolic reprogramming in OS cells. However, the m^6^A methylated sites on the ERRα 3′-UTR require further investigation.

The present study suggested that the IGF2BP1-regulated ERRα expression could be involved in metabolic reprogramming in OS cells resistant to chemotherapy. Mechanistically, IGF2BP1 could bind with the m^6^A methylated 3′-UTR of ERRα mRNA to increase its mRNA stability, thus leading to enhanced glycolysis and resistance to Dox. The above findings indicated that targeting the IGF2BP1/ERRα axis-induced glucose metabolism could be considered as a potential strategy for overcoming Dox-resistance in OS cells.

## Materials and methods

### Cell line, cell culture and treatment

The human OS cell lines, namely MG-63 and HOS, were purchased from the Chinese Academy of Sciences. Dox-resistant OS cells were established by culturing OS cells with increasing concentrations of Dox for approximately 6 months, as previously described [9]. The Dox-resistant OS cell lines were named MG-63/Dox and HOS/Dox cells. The half maximal inhibitory concentration of Dox in MG-63/Dox and HOS/Dox cells was 7.56 and 9.25 μM, respectively. All cells were cultured in Dulbecco's modified eagle medium (DMEM) containing 10% fetal bovine serum, penicillin and streptomycin. Dox-resistant cells were incubated with DMEM containing 0.5 μM Dox to maintain chemoresistance. The medium supplemented with Dox was replaced with complete medium three days prior each experiment. XCT-790, a specific inverse ERRα agonist, and other chemicals were of analytical grade or higher and were all purchased from Sigma-Aldrich Co. LLC unless otherwise noted. All compounds were solubilized in dimethyl sulfoxide (DMSO), while medium supplemented with 0.5% v/v DMSO served as control.

### Measurement of ATP, lactate and glucose consumption

The measurement of ATP, lactate and glucose consumption was performed using the corresponding commercial kits according to manufacturer's instructions and previous studies [35, 36]. Briefly, a bioluminescent ATP assay kit (cat. no. #S0027; Beyotime Institute of Biotechnology) was used for measuring ATP levels. ATP levels in each well were measured on a multi-mode reader (BioTek Instruments, Inc.), calculated using a calibration curve and normalized to total protein levels.

2-NBDG (MilliporeSigma), a fluorescent glucose analog, was utilized to measure glucose levels. Briefly, following treatment, cells were incubated with medium containing 10 μM 2-NBDG for 30 min. The fluorescence intensity of 2-NBDG was detected using a multi-mode reader (BioTek Instruments, Inc.). The excitation and emission wavelengths were 494 and 551 nm, respectively. Lactate levels in culture medium were determined using the Lactate Assay Kit (cat. no. #L256; BioVision, Inc.). Similar to ATP, the levels of lactate were calculated using a calibration curve and its levels were normalized to those of total proteins.

### OCR and ECAR

OCR was determined using the Seahorse XF96 Extracellular Flux (Seahorse Bioscience) according to the manufacturer’s protocol as previously described [37]. The DMEM XF Assay media, XF96 sensor cartridges, XF96-well plates and calibration buffer was obtained from Seahorse Bioscience. Briefly, cells were seeded into a Seahorse cell plate and cultured until attached. Subsequently, cells were treated with 1 μM specific electron transport chain inhibitors, including oligomycin, FCCP and rotenone/antimycin A. The real-time OCR was measured and normalized to protein concentration. ECAR was calculated under basal conditions and was increased after the addition of glucose that provided glycolytic flux. In addition, oligomycin was used to measure glycolytic capacity.

### Western blot analysis

Following lysis with radioimmunoprecipitation assay buffer (MilliporeSigma) supplemented with protease inhibitors (Roche Diagnostics), equivalent proteins (20 μg) were separated by 10% SDS-PAGE. Subsequently, proteins were transferred to a PVDF membrane (MilliporeSigma) and were then blocked with 5% skim milk in PBS-Tween 20. The membrane was incubated with the following primary antibodies (dilution, 1:1000) at 4 °C overnight: Anti- GAPDH (cat. no. ab181602; Abcam), anti-ERRα (cat. no. sc‐65718; Santa Cruz Biotechnology, Inc.) and anti-IGF2BP1 (cat. no. 22803-1-AP; ProteinTech Group, Inc.). Following washing to remove primary antibodies, the membranes were incubated with the corresponding secondary horseradish peroxidase (HRP)-conjugated IgG antibody (dilution, 1:5000) at room temperature for 90 min. Enhanced chemiluminescence (ECL; Beyotime Institute of Biotechnology) was used to visualize the protein bands with the BioImaging System (GE Healthcare). The expression levels of ERRα and IGF2BP1 were normalized to those of GAPDH.

### Cell transfection

The negative control siRNA (si-NC) and siRNAs targeting ERRα were obtained from Guangzhou RiboBio Co., Ltd. The ERRα cDNA was digested with BamHI/EcoRI restriction enzymes and was then subcloned into the pcDNA3.1 vector to construct the pcDNA-ERRα overexpression plasmid. Cells were transfected with the above plasmids/siRNAs using Lipo3000 (Invitrogen; Thermo Fisher Scientific, Inc.) according to manufacturer's instructions. To generate stable ERRα-depleted OS cells, cells were transfected with control or ERRα lentiviral shRNAs and were then selected using puromycin.

### Reverse transcription-quantitative PCR (RT-qPCR)

To determine the mRNA expression levels of target genes, real time PCR was carried out as previously described [9, 38]. The primer sequences used were as follows: For ERRα, forward, 5′-GAG ATC ACC AAG CGG AGA CG-3′ and reverse, 5′-ATG AGA CAC CAG TGC ATT CAC-3′; for β-actin, forward, 5′-CAT GTA CGT TGC TAT CCA GGC-3′ and reverse, 5′-CTC CTT AAT GTC ACG CAC GAT-3′; for F-Luc, forward, 5′-CGA GGC TAA GGT GGT GGA CTT GG-3′ and reverse, 5′-CCA GCC GTC CTT GTC GAT GAG AG-3′; and for R-Luc, forward, 5′-TGT CCG CAA CTA CAA CGC CTA CC-3′ and reverse, 5′-GCG TCC TCC TGG CTG AAG TGG-3′. The primers crossing exon 1 and the following intron were used to measure the expression levels of precursor ERRα mRNA. The primer sequences used were: Forward, 5′-GCG ATG TCC TTT TGT GTC CT-3′ and reverse, 5′CCT GAA CCC TGA CCA GTC C-3′. The mRNA expression levels were normalized to the relative expression levels of β-actin using the 2^−ΔΔCq^ method [39].

### Dual luciferase assay

The transcriptional activity of ERRα promoter was assessed by dual luciferase assay as previously described [9, 38]. The ERRα promoter region (− 1000 to − 1 bp) was subcloned into the luciferase promoter to construct the pTL-ERRα plasmid. Cells were transfected with both pTL-ERRα and pBABE-puro plasmids and the relative promoter activity was determined by normalizing the activity of F-Luc to that of R-Luc. The pmirGLO plasmid was used to evaluate the potential effect of ERRα 3′-UTR on F-Luc mRNA stability. Briefly, the CDS or 3′-UTR of ERRα mRNA (Accession Number, NM_004451.5) was subcloned into the pmirGLO vector using FastDigest restriction enzymes (Thermo Scientific, Inc.) with specific primers between the SacI and SalI restriction sites, as previously described [40]. The correct sequence was verified by DNA sequencing using a specific forward 3′-end luciferase primer. Following transfection with pmirGLO-CDS or pmirGLO-3′-UTR for 24 h, the activity of F- and R-Luc was measured by dual luciferase assay. The relative mRNA expression levels of F-Luc and R-Luc were measured using RT-qPCR.

### mRNA stability assay

For mRNA stability assay, cells were cultured in complete medium containing 5 μg/ml Act-D (MilliporeSigma) to block mRNA transcription. Following incubation for 0–8 h, total RNA was extracted and the mRNA expression levels of ERRα were then detected.

### Cell proliferation analysis

A Cell Counting Kit-8 (CCK-8) assay was carried out to evaluate cell proliferation as previously described [9].

### ***m***^***6***^***A-RIP qPCR***

m^6^A-RIP qPCR was performed as previously described [41]. Briefly, 200 μg extracted RNA was incubated with Protein G Magnetic beads-coated with m^6^A or IgG antibody at 4 °C for 3 h in 1 × Reaction buffer (150 mM NaCl, 10 mM Tris–HCl, pH 7.5, 0.1% NP-40 in nuclease free H_2_O). The bounded RNA was eluted following incubation with 100 μl Elution Buffer [75 nM NaCl, 50 nM Tris–HCl, pH 7.5, 6.25 nM EDTA, 1% (w/v) SDS, 20 mg/ml Proteinase K] for 30 min at room temperature. RNA was then extracted by a standard phenol/chloroform method. After RT, the relative enrichment of the target mRNA was measured by qPCR. The immunoprecipitation (IP) enrichment ratio of a transcript was determined as the ratio of its amount in IP to that in the input yielded from same amounts of cells.

### RIP-RT-PCR

Two 10-cm plates of cells were irradiated twice with 400 mJ/cm^2^ at 254 nm by Stratalinker on ice, and then washed twice with cold PBS before collected. 400 μl IP lysis buffer [150 mM KCl, 25 mM Tris (pH 7.4), 5 mM EDTA, 0.5 mM DTT, 0.5% NP40, 1 × protease inhibitor, 1 U/μl RNase inhibitor] was added and resuspended at 4 °C for 30 min. The lysate was centrifuged at 12,000*g* for 10 min. Then Magnetic beads pre-coated with 4 μl targeted antibodies or mouse IgG (NEB, USA) were incubated with sufficient cell lysates with RNase inhibitors at 4 °C overnight. The beads containing immunoprecipitated RNA–protein complex were treated with proteinase K to remove proteins. Then RNAs were purified by with phenol: chloroform extraction followed by ethanol precipitation. The interested targets were detected by RT-qPCR with the same primers. Antibodies included anti-YTHDF2 (ab220163, Abcam), anti-IGF2BP1 (8482S, Cell Signaling), anti-IGF2BP2 (14672S, Cell Signaling), anti-IGF2BP3 (25864S, Cell Signaling), and anti-IgG (ab48386, Abcam).

### Tumorigenesis assay

Nude mice were purchased from the Sun Yat-sen University Animal Center (Guangzhou, China) and maintained under pathogen-free conditions. Animal study was approved by Sun Yat-sen University School of Medicine Animal Care and Use Committee (SYSU-IACUC-2021-B). All animal studies were conducted in accordance with the institutional guidelines for the Care and Use of Experimental Animals. MG-63, MG-63/Dox or IGF2BP1-depleted MG-63/Dox cells (2 × 10^6^ cells/mouse) in 50% Matrigel solution (BD Bioscience) were subcutaneously injected into the fourth right mammary fat pad at the base of the nipple of nude mice (n = 5). When the tumor was visible, mice were treated by tail vein injection of 3 mg/kg Dox with or without 2 mg/kg XCT-790 for four times every three days. Mice in the control group were treated with an equal volume of vehicle. Tumor growth and body weight were monitored every three days. The tumor volume was calculated using the following formula: $${\text{V}} = {\raise0.7ex\hbox{$1$} \!\mathord{\left/ {\vphantom {1 2}}\right.\kern-\nulldelimiterspace} \!\lower0.7ex\hbox{$2$}} \times {\text{larger}}\,{\text{diameter}}\, \times \left( {{\text{smaller}}\,{\text{diameter}}} \right)^{2}$$. At the end of the treatment period, the animals were sacrificed and the tumors were removed and weighed.

### Bioinformatics analysis

The mRNA expression levels of IGF2BP1 and ERRα in OS and normal tissues were analyzed in the Oncopression database (https://www.oncopression.com) as previously described [42], followed by further analysis and visualization using GraphPad Prism software. The Kaplan–Meier database (http://kmplot.com/analysis/) was used to determine the association between overall survival and IGF2BP1 and ERRα expression. The differences between survival curves were compared by log-rank test and P < 0.05 was considered to indicate as statistically significant difference. The correlations between IGF2BP1 and ERRα were extracted from TCGA and Oncomine (www.oncomine.org) databases. The data were then analyzed by Pearson Chi-square (χ2) test.

### Statistical analysis

Numbers of replications per experiment are indicated in the figure legend. Each experiment was repeated three times. All data are expressed as the mean ± standard deviation (SD). The differences between two groups were assessed by two-tailed Student’s t-test, while those among multiple groups by one-way ANOVA. P < 0.05 was considered to indicate a statically significant difference.

## Data Availability

All data generated or analyzed during this study are included in this published article.
